# Treatment of cerebrospinal fluid leakage with prolonged use of subfascial epidural drain and antibiotics in patients of thoracic myelopathy after posterior decompression surgery

**DOI:** 10.3389/fsurg.2023.1302816

**Published:** 2023-11-16

**Authors:** Jiliang Zhai, Shigong Guo, Da He, Yu Zhao

**Affiliations:** ^1^ Department of Orthopaedic Surgery, Peking Union Medical College Hospital, Chinese Academy of Medical Science and Peking Union Medical College, Beijing, China; ^2^ Department of Rehabilitation Medicine, Southmead Hospital, Bristol, United Kingdom; ^3^ Spine Department, Beijing Jishuitan Hospital, Beijing, China

**Keywords:** cerebrospinal fluid leakage, thoracic spinal stenosis, thoracic myelopathy, ossification of ligamentum flavum, dura ossification

## Abstract

**Background:**

Cerebrospinal fluid leakage (CSFL) is a prevalent and vexing complication associated with spine surgery. No standard protocol is available guiding CSFL management, especially for thoracic CSFL. The aim of this study was to retrospectively evaluate the efficacy of prolonged use of subfascial epidural drain and antibiotics to treat CSFL after posterior thoracic decompression surgery.

**Methods:**

Fifty-six patients with an average age of 52.3 years (24–76 years), who underwent thoracic decompression with CSFL (group A) and 65 patients with an average age of 54.9 years (25–80 years) without CSFL (group B) were retrospectively reviewed. Patients in group A had prolonged use of subfascial drainage and antibiotics and patients in group B were treated with conventional methods. The surgical results and rate of wound related complications was compared between the two groups.

**Results:**

The average subfascial drainage time was 7.0 ± 2.7 days (2–16 days) and 3.8 ± 1.4 days (2–7 days) in group A and B, respectively. Higher occupation rate (>49%), presence of dural ossification and higher MRI grade (>2) were more likely to presented with CSFL. In group A, four patients (7.1%) presented with deep wound infection and were successfully managed with wound debridement or intravenous antibiotics. In group B, one patient (1.5%) had a superficial wound infection and was treated with antibiotics. No patients presented with wound dehiscence, wound exudation or CSF fistulation.

**Conclusion:**

The occupation rate of ossified mass and presence of dural ossification were the major risk factors of CSFL. No significant difference in infection rates was observed between the patients in group A and B.

## Introduction

Cerebrospinal fluid leakage (CSFL) is a prevalent and vexing complication associated with spine surgery, identified by intraoperative detection of dura tear or postoperative occurrence of clear drainage fluid outflow (1). Reported rates of CSFL ranged from <1% to 17% for spine surgeries (2, 3). The incidence of CSFL in primary lumbar spine surgeries ranged from 5.5% to 9% and was as high as 13.2% to 21% in lumbar revision surgeries (4). The occurrence of inadvertent durotomies subsequent to anterior cervical decompression procedures varied between 0% and 8.3%, with a notable rise observed in patients diagnosed with ossification of the posterior longitudinal ligament (OPLL), ranging from 4.3% to 32% (5–7). There were few reports about CSFL after thoracic decompression surgery, and the incidence varied from 10% to 22.2% (8–10).

Persistent CSFL had been linked to a range of detrimental consequences, including intracranial hypotension, fistula, wound infection, intracranial hemorrhage, arachnoiditis, nerve root ncarceration/strangulation and meningitis (11–14). It also increased the health-care expenses, with an average of $6,479 per patient in the United States of America compared to the patients who did not have CSFL (15). However, to the best of our knowledge, no standard protocol is available guiding CSFL management, especially for thoracic CSFL. In a prospective study on subfascial drainage for CSF after posterior spine surgery, Fang et al. (1) showed that subfascial drainage for more than 7 days had a lower rate of complications than that for less than 7 days. However, treatment of thoracic CSFL with prolonged use of subfascial epidural drain has not been reported before. The objective of this study was to retrospectively review the efficacy of prolonged use of subfascial epidural drain and antibiotics in the treatment of thoracic CSFL and compare the results with the patients without CSFL with regular treatment.

## Methods

### Patients

From September 2012 to September 2021, 121 patients who underwent thoracic decompression surgery due to thoracic myelopathy caused by ossification of ligamentum flavum (OLF) or OPLL were retrospective reviewed. No patients were enrolled and formal consent was not required in this study, which was approved by the institutional review board of our hospital (NO. K3408). Patients with infection, tumor, fracture, and spinal deformity were excluded from this study. 56 patients (21 male and 35 female) with CSFL were classified into group A, and 65 patients (36 male and 29 female) without CSFL were classified into group B. The average age at surgery was 52.3 ± 11.2 years (24–76 years) and 54.9 ± 11.9 years (25–80 years), respectively. All patients underwent primary surgery except five patients in group A and four in group B underwent revision surgery. Demographic data, surgical information, perioperative CSFL management, clinical and radiographic features, and complications were retrieved from patients' medical records. Patients were followed up regularly in the outpatient clinic. Neurological function was evaluated with Japanese Orthopaedic Association (JOA) score and neurologic recovery rate was calculated as = (final JOA—preoperative JOA)/(17- preoperative JOA) × 100% (16). Tram track sign (TTS), comma sign (CS) and bridge sign (BS) on axial CT image and intraoperative confirmation of the dura ossification (DO) were recorded. The thickness of the ligament ossified mass was measured using Image J software (National Institutes of Health, Bethesda, MD, USA). The spinal canal encroachment rate of the ossified mass was represented by anteroposterior diameter ratio (APDR) for OPLL or unilateral diameter ratio (UDR) for OLF. APDR was defined as the ratio of anterior to posterior diameter of OPLL mass to that of spinal canal at the same level on CT axial image. UDR was measured as the ratio of the maximum thickness of OLF mass from top to bottom to that of spinal canal from central posterior margin of the vertebral body to the bottom of lamina (17). The degree of spinal canal compression was divided into four levels on axial T2-weighted MRI (18).

### Surgical procedure

All surgical procedures were performed by a single senior spine surgeon. Thoracic laminectomy and ligamentum flavum resection were performed in all cases. For patients with OPLL as the main pathogenic factor, circular decompression was performed. The ossified dura mater was also resected if presented. For patients in group A, two underwent direct repair of dural tear and others underwent indirect repair because the dural defect was too big or located too far out to adopt direct repair. A piece of hemostatic sponge or gelatin sponge was layered over the durotomy site, followed by layered wound closure in indirect repair. For all the patients, a drainage tube was placed under the muscular layer, connected to a 1,000 ml bag without suction to establish a closed drainage system. The crucial deep fascial layer was closed through a single continuous suture, with intermittent reinforcement sutures to strengthen the closure.

### Postoperative treatment

Postoperative management was different between group A and B. In group A, the patients laid supine in the Trendelenberg position postoperatively with the head down to 15°. The drainage collection bag was kept below the patient's bed to avoid over-drainage of CSF. CSF flow was monitored to ensure the drainage was around 300 ml every 24 h and to detect early signs of infection. If increased drainage was observed, the drainage bag height level was raised to reduce drainage volume. The drainage tube was removed when the drainage less than 50 ml within 24 h or 7 days after operation. The drain tube tract was closed with a figure-of-eight suture to prevent CSF fistulation. Patients could get up and walk after removal of the drainage tube. Prophylactic intravenous vancomycin and third-generation cephalosporin were prescribed postoperatively until drainage tube was removed and body temperature was normal. For patients with fever, Zyvox and meropenem were used to replace vancomycin and third-generation cephalosporin. For patients in group B, the same drainage system with group A were used. The patients were bedridden in supine for 2–3 days due to pain or other discomfort. Drainage tube was removed when the amount of drainage was less than 50 ml within 24 h. Second-generation cephalosporin was administered for less than 24 h postoperatively.

### Statistical analysis

The Statistical Package for Social Sciences (SPSS Inc., Chicago, IL, USA) software was used for data analysis. The enumeration data and measurement data were analyzed by *χ*^2^ and *t*-test, respectively. Logistic regression analysis was used to determine the risk factors of infection rate and CSFL. *P* value < 0.05 indicates statistical significance.

## Results

### Demographics

Demographic data and clinical features were shown in Table 1. No significant difference was observed regarding age, body mass index (BMI), number of operated segments, blood loss and length of hospital stay between patients in groups A and B. The average subfascial drainage time was 7.0 ± 2.7 days (2–16 days) and 3.8 ± 1.4 days (2–7 days) in groups A and B, respectively, which was significantly different in both groups. In terms of the preoperative, postoperative, and final follow-up JOA scores, Group A exhibited significantly lower scores compared to Group B. Nevertheless, no significant difference was found in the rate of neurological recovery between the two groups.

**Table 1:** Demographic data and clinical features of the patients with or without CSF leak.

	Group A	Group B	*p* value
Gender (M/F)	21/35	36/29	/
Age (years)	52.3 ± 11.2	54.9 ± 11.9	0.213
BMI (Kg/m^2^)	28.0 ± 4.1	27.5 ± 5.7	0.592
Number of operated segments	5.5 ± 2.1	4.8 ± 2.3	0.092
Blood loss (ml)	1,053.6 ± 700.1	893.8 ± 742.0	0.226
Drainage time (days)	7.0 ± 2.7	3.8 ± 1.4	0.000
Length of hospital stay (days)	19.1 ± 10.5	26.9 ± 63.0	0.361
Follow-up time (months)	67.9 ± 33.1	62.3 ± 35.0	0.370
JOA score			
Pre-operative	9.8 ± 2.9	11.5 ± 2.5	0.001
Post-operative	11.3 ± 3.3	13.1 ± 1.2	0.001
At final follow-up	13.3 ± 3.5	14.6 ± 2.7	0.018
Recovery rate (%)	57.1 ± 36.0	59.7 ± 41.2	0.717

BMI, body mass index; JOA score, Japanese orthopaedic association score.

**Figure 1:**
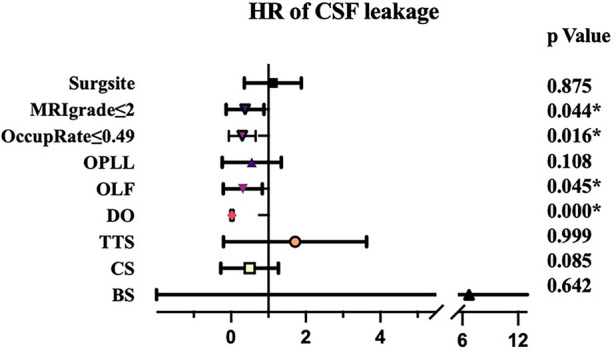
Logistic regression analysis results about risk factors of cerebrospinal fluid leakage.

Radiological features of the patients were shown in Table 2. OLF was main reason for surgery for both two groups (98.2% and 86.2%, respectively) and most of the patients underwent surgery at lower or middle thoracic vertebra. TTS, CS, BS and DO were significant more prevalent in group A than that in group B. Moreover, the spinal canal encroachment rate caused by the ossified mass was significantly higher in Group A (53.4% on average) compared to Group B (41.5%). As for MRI grading, the ratio of Grade IV was also significantly higher in group A. Logistic regression analysis showed that patients with higher occupation rate (>49%), presence of DO and higher MRI grade (>2) were more likely to presented with CSFL (Figure 1).

**Table 2:** Radiological features of the patients with or without CSF leak.

	Group A	Group B	*p* value
Surgical site			
Upper thoracic vertebra (T1–4)	12	10	0.480
Middle thoracic vertebra (T5–8)	15	6	0.015
Lower thoracic vertebra (T9–12)	20	38	0.017
Thoracic and lumbar vertebrae	7	11	0.611
Cervical and thoracic vertebrae	2	0	0.212
Thoracic OPLL	21	12	0.024
Thoracic OLF	55	56	0.020
Tram track sign	25	17	0.037
Comma sign	8	3	0.023
Bridge sign	10	2	0.064
DO	50	10	0.000
APDR/UDR (%)	53.4 ± 20.9	41.5 ± 16.4	0.001
MRI grading			
1	0	1	1.000
2	4	15	0.023
3	5	13	0.039
4	47	36	0.000

OPLL, ossification of posterior longitudinal ligament; OLF, ossification of ligamentum flavum; DO, dura ossification; APDR, anteroposterior diameter ratio; UDR, unilateral diameter ratio.

### Complications

One of two patients in group A who underwent direct repair still presented with CSFL postoperatively. One patient experienced hemorrhagic shock and complete paralysis of lower limbs due to spinal cord ischemia in group A. Partial recovery was achieved through rehabilitation and physical therapy exercises. Another two patients had spinal cord injury and weakness of the lower limbs with partial recovery after rehabilitation and two patients were found to have malposition of the screw and underwent revision surgery. In group B, two patients had temporary weakness of the lower limbs and one patient underwent revision surgery due to implant failure.

Twelve patients had symptoms of intracranial hypotension in group A, including dizziness in 10, photophobia in 6 (4/6 had dizziness), and headache in 4 patients (4/4 had dizziness). In group A, none had incisional CSFL. However, one patient underwent debridement due to delayed wound healing. Four patients (7.1%) suffered from deep wound infection. Among them, one patient underwent debridement, while the remaining three cases were successfully managed with antibiotic treatment, leading to a smooth recovery for all four patients. In group B, one patient suffered from superficial wound infection (1.5%) and was treated with antibiotics. No patients in either group exhibited wound breakdown, wound exudation, or cerebrospinal fluid fistulation. The infection rates between the two groups did not display a significant difference (*P *= 0.122). And infection was not related to age, BMI, number of operated segments, occupation rate of ossified mass, blood loss, drainage time and presence of CSF.

## Discussion

### The rate of cerebrospinal fluid leakage after spine surgery

Durotomy-induced CSFL is undesirable but relatively common in spine surgery, especially in cases with dural adhesion and dural ossification. The incidence varied among different procedures and different series of patients (19). For instance, Cammisa et al. (20) conducted a retrospective study of 2,144 patients, including 422 cervical surgery (338 anterior and 84 posterior), 7 posterior thoracic surgery, and 1,715 lumbosacral surgery (1,646 posterior and 69 anterior), and the overall incidence of CSFL was 3.5%. In another study, Woff et al. (21) observed that 1.7% of 1,359 lumbar patients had CSFL. However, Khan et al. (22) reported an overall CSFL incidence as high as 10.6% in 3,183 lumbar patients, which consisted of the largest number of cases. Hannallah et al. (23) noted that 1% of 2,216 cervical spine procedures had CSFL. In a retrospective review of 362 cases of thoracic decompression surgery, the incidence of CSFL after thoracic spinal surgery was reported to be 32.3%, and different surgical approaches had different incidences of CSFL (24). In a systematic review of thoracic spine surgery for OLF, the incidence of CSFL was 19% (25), and it was reported to be 22.5% for patients with OPLL in another systematic review (26). Generally, CSFL was found to be more common in thoracic surgery than that in lumbar and cervical spine surgery. In a previous study, older than 52 years, OPLL and longer than 3 operative vertebrae were significant risk factors for CSFL and surgeries on the mid-thoracic spine increased the risk of CSFL (24). However, in our study, the incidence of CSFL was not related with the number of operative segments, surgical site and presence of OPLL, TTS or CS. The major risk factors of CSFL were the occupation rate of ossified mass and presence of DO.

### Repair strategy for dural tear

CSFL can lead to various complications if not managed properly, including non-healing wounds, infections, CSF fistulas, and meningitis. The treatment of CSFL can be classified into two treatment regimens: (1) directly close with sutures or indirectly close with the onlay technique using dural substitute material to stop CSFL; (2) reduce the subarachnoid fluid and/or increase the epidural space pressure to decelerate CSFL (19).

The goal of surgical repair of dural tears is to produce an adequate seal that can withstand CSF pressure during the healing period. Generally, direct repair is the best way to treat dural tear (27). The success rate is up to 70% in cervical and lumbar spinal surgeries. However, the success rate is lower, around 30%, in thoracic decompression procedures due to the vulnerability of the dura in the thoracic spine and the irregular nature of the breaches (28). Besides, evidence showed that repair with primary dural closure is not always necessary (4). Some surgeons choose not suturing if there is no breach in the arachnoid (29, 30). The rationale for this approach was that the risk of arachnoid herniation is balanced by the risk of CSFL through the needle holes during suturing. In addition, direct suture repair may increase the operative time and surgical risk (1). Indirect closure with the onlay technique will be indicated when it is impossible to suture directly onto the edge of the dura mater or dura tear that involves the nerve root sleeve or axilla, or if dural tear lies anteriorly, presence of a large dural defect, or poor tensile strength of the dura. Fat/fascia/muscle grafting, synthetic grafts, and collagen matrix/gelatin sponge can be used for this technique (29). Therapy with indirect repair was successfully carried out in patients with CSFL. Brazdzionis et al. (31) retrospectively enrolled 21 patients with incidental durotomy. Nine of them underwent direct suture of the dural tear and 12 indirectly repaired with a sealant. No patients developed CSF fistulas in both groups and length of hospital stay and infection rate did not differ between the two groups. Comparing the advantages and disadvantages of direct and indirect repair, all except two patients underwent indirect repair in our study and recovered smoothly without serious complications.

### The role of prolonged wound drainage

The time of wound drainage is a controversial topic among surgeons. Some surgeons argue in favor of controlled continuous drainage to prevent meningoceles and extradural hematomas (32). For patients with CSFL, continuously evacuating CSF out of the wound, has been shown to facilitate sufficient healing and sealing time for the dura, soft tissue, and fascia, thereby preventing dead space (33) and promoting epithelialization of the surgical wound, which helps prevent the formation of CSF fistulas (12). Some researchers counter the placement of drainage due to concern of CSF hypovolemia due to overdrainage (34), which will induce headache, nausea, and vomiting (30). There was also a concern of complications associated with closed wound drains, including infection, hematoma formation, and additional neurological deficit (35, 36).

Conventionally, the drain tube was removed when the drainage output turned clear. However, this is not universally accepted (33). Some propose leaving the drainage in place for a longer duration than usual, but there is no consensus on the optimal duration of subfascial drainage. In a retrospective study of 266 patients with thoracic myelopathy caused by OLF, 65 patients had CSF leakage postoperatively. The subfascial drain was removed when the drainage reduced to less than 50 ml per 24 h or the color of drainage fluid became clear. The latter situation required other comprehensive treatment, where the patient was placed in a prone position with a sandbag on the wound to give continuous pressure. After 5–7 days in this position, the patients were mobilized. Sixteen patients failed after this algorism, including 4 wound dehiscence, 2 infection and 10 pseudocyst (37). Hughes et al. (33) proposed this time to be about 10–17 days postoperatively for patients without suture of durotomy. Fang et al. (19) recommended drain tube duration of more than 7 days. Others support postoperative drainage for an average of 3 days (1). In the process of soft tissue repair, the inflammatory response starts in 2 days. Primary fibroblastic bridging occurs until postoperative day 6, and the surface is coated with inflammatory cells until postoperative day 10 (36, 38). Considering the above results, we left the wound drain in-situ for an average of 7.6 days in group A in our study. The positive results showed that none of the patients suffered from wound breakdown or fistula.

### The role of preventive use of antibiotics for cerebrospinal fluid leakage after spine surgery

Another risk of wound drains is ascending infection or meningitis from a tube left in place for a long time. The rate of deep wound infection could be as high as 8.1% in patients with durotomies (20). Although there is a consensus on the pertinence of prophylactic antibiotic therapy at induction (39, 40), the indication for prolonged antibiotic therapy, when a tear occurs, is subject to debate (21). The use of prophylactic antibiotics is necessary, and debridement should be performed with no hesitation to avoid the spread of infection into the central nervous system. In a study of 65 patients with CSFL, 4 had wound dehiscence, 2 suffered from infection, and one was dead from the central nervous system infection (37). Considering the risk of infection and secondary serious consequences such as meningitis and death, we adopted prolonged use of antibiotics, which had good penetration in CSF, to cover common organisms (41). The rate of infection had no significant difference between the patients in group A and group B, and no serious complications such as meningitis and death occurred in group A, suggesting the safety and efficacy of prolonged use of antibiotics.

## Limitations

Several limitations of this study should be noted. This was a retrospective study and had all the limitations of retrospective studies. There was no control group that had a CSFL without prolonged use of drainage and antibiotics. However, it is clinically not suitable to set up this control group due to the high risk of infection, and serious adverse consequences.

## Conclusions

Higher occupation rate, presence of DO and higher MRI grade were the risk factors for CSFL after thoracic spinal decompression surgery. There were no significant differences of infection rate or wound complications between the patients of CSFL with prolonged use of subfascial drainage and patients without CSFL. Prolonged subfascial epidural drainage and antibiotics can effectively manage CSFL when faced with thoracic dura tear.

## Data Availability

The raw data supporting the conclusions of this article will be made available by the authors, without undue reservation.
